# Antidiabetic Effect of Taxifolin in Cultured L6 Myotubes and Type 2 Diabetic Model KK-A^y^/Ta Mice with Hyperglycemia and Hyperuricemia

**DOI:** 10.3390/cimb43030092

**Published:** 2021-09-26

**Authors:** Shinji Kondo, Shin-ichi Adachi, Fumiaki Yoshizawa, Kazumi Yagasaki

**Affiliations:** 1 Center for Bioscience Research and Education, Utsunomiya University, Utsunomiya 321-8505, Japan; kondo.shinji.ga@u.tsukuba.ac.jp (S.K.); adachi@tamateyama.ac.jp (S.-i.A.); 2 School of Agriculture, Utsunomiya University, Utsunomiya 321-8505, Japan; fumiaki@cc.utsunomiya-u.ac.jp; 3 United Graduate School of Agricultural Science, Tokyo University of Agriculture and Technology, Tokyo 183-8509, Japan

**Keywords:** taxifolin, L6 myotube, PI3K, Akt, AMPK, GLUT4, type 2 diabetes, KK-A^y^/Ta mouse, hyperglycemia, hyperuricemia

## Abstract

Muscle is the largest tissue in our body and plays an important role in glucose homeostasis and hence diabetes. In the present study, we examined the effects of taxifolin (TXF) on glucose metabolism in cultured L6 muscle cells (myotubes) and in type 2 diabetic (T2D) model KK-A^y^/Ta mice. TXF dose-dependently increased glucose uptake (GU) in L6 myotubes under the condition of insulin absence. This increase in GU was partially, but significantly canceled by TXF treatment in combination with either LY294002, an inhibitor of phosphatidylinositol 3-kinase (PI3K), which phosphorylates protein kinase B (Akt) or Compound C, an inhibitor of 5’-adenosine monophosphate-activated protein kinase (AMPK). Furthermore, TXF was demonstrated to activate (=phosphorylate) both Akt and AMPK, and promote glucose transporter 4 (GLUT4) translocation to the plasma membrane from cytosol of L6 myotubes via both PI3K/Akt and AMPK signaling pathways. Based on these in vitro findings, we conducted an in vivo experiment in KK-A^y^/Ta mice with hyperglycemia and hyperuricemia. Fasting plasma glucose, insulin, uric acid levels and an index of insulin resistance (HOMA-IR) increased significantly in the T2D model mice compared with normal ones. Such rises in the T2D state were significantly suppressed by oral administration of TXF for four weeks. These results suggest that TXF is a potent antihyperglycemic and antihyperuricemic phytochemical in the T2D state.

## 1. Introduction

The number of patients with diabetes is increasing worldwide and the global diabetes prevalence in 2019 has been estimated to be 463 million people, rising to 578 million by 2030 and 700 million by 2045 [1]. There are three main types of diabetes, namely, type 1 diabetes (T1D), type 2 diabetes (T2D), and diabetes in pregnancy (DIP). Of these, T2D accounts for approximately 90% of the total [1,2], and this rising trend can be attributed to aging, urbanization, and the obesogenic environment [2]. Skeletal muscles constitute one of the largest tissues in the body, and contribute to metabolic regulations of the main nutrients such as proteins/amino acids, lipids, and glucose. In glucose metabolism, the skeletal muscles account for the majority (~75%) of insulin-mediated glucose uptake in the post prandial state, and hence plays an important role in maintaining glucose homeostasis [3]. In protein metabolism, leucine is unique among the branched-chain amino acids (BCAAs) in its ability to stimulate protein synthesis in the muscle of food-deprived rats [4]. L6 myoblasts established from rat skeletal muscle tissues are well known to grow rapidly, fuse spontaneously, differentiate into multinucleated myotubes [5], and are widely used in the studies on the effects of various biofactors on muscle functions in vitro. The stimulatory effect of leucine on protein synthesis has been demonstrated in differentiated L6 myocytes (myotubes) [6,7] through several signaling pathways. Leucine has been also demonstrated to stimulate glucose uptake (GU) in the soleus muscle of rats [8]. From the aspects of screening and studying mechanisms of biologically active compounds, cultured cells of established lines have been recognized to be useful, for example, studies on anticancer biofactors [9]. Thus, we contrived a simple, rapid, and inexpensive assay system for GU under the condition of insulin absence by adopting cultured L6 myotubes [10]. Aspalathin, a rooibos tea component, was the first phytochemical that we could find in this simple assay system to increase GU in L6 myotubes under the condition of insulin absence and to show antihyperglycemic effect and its mechanisms in T2D model db/db and ob/ob mice [10,11]. Thus far, we have found several effective phytochemicals such as aspalathin, resveratrol, piceatannol, nepodin, gingerol, genistein, daidzein, equol, and enterolactone [12,13,14]. Of these, equol and enterolactone are metabolites of daidzein and lignans, respectively, by enterobacteria [14]. These phytochemicals and their intestinal metabolites promoted the 5’-adenosine monophosphate-activated protein kinase (AMPK) pathway and/or protein kinase B (Akt) pathway, thereby stimulating the translocation of glucose transporter 4 (GLUT4) to the plasma membrane of L6 myoblasts or L6 myotubes under the condition of insulin absence, leading to the attenuation of hyperglycemia in T2D model db/db, ob/ob and KK-A^y^/Ta mice, even under the condition of insulin resistance [12,13,14].

Taxifolin (TXF) or dihydroquercetin is widely found in various plants [15] including edible ones such as strawberry [16], and has been found to show various pharmacological activities such as antioxidant, anti-inflammatory, antihyperlipidemic, antiangiogenic, anticancer, antimicrobial [15], and antidementia activities [17].

Meanwhile, Zhu et al. demonstrated that high uric acid (UA) directly inhibits insulin signaling and induces insulin resistance [18]. Adachi et al. found that the levels of plasma uric acid, glucose, insulin, homeostasis model assessment of insulin resistance (HOMA-IR), and triglyceride in KK-A^y^/Ta mice were significantly higher than those in normal mice. In addition, plasma uric acid levels showed significant and positive correlations with plasma glucose, insulin, HOMA-IR and triglyceride levels [19]. TXF has been demonstrated to suppress UA production in AML12 hepatocytes and purine body-induced hyperuricemia in mice [20].

We have recently found a possibility that TXF promotes GU in cultured L6 myotubes under the condition of insulin absence. In the present study, we confirmed this possibility by investigating the dose-responses and modes of action of TXF on GU in L6 myotubes in vitro and examined the effects of TXF on hyperuricemia as well as hyperglycemia in T2D model KK-A^y^/Ta mice in vivo. Results demonstrate that TXF is a potent anti-hyperglycemic and anti-hyperuricemic phytochemical.

## 2. Materials and Methods

### 2.1. Evaluation of Glucose Uptake in L6 Myotubes

L6 myoblasts were provided from American Type Culture Collection (ATCC^®^, CRL-1458, Lot No. 61875400. Manassas, VA, USA) and grown in Dulbecco’s modified Eagle’s medium (DMEM, Cat No. 05919, Lot No. 07570461, Nissui Pharmaceutical Co., Tokyo, Japan). The medium was supplemented with 10% (*v/v*) fetal bovine serum (FBS) (Cat No. SH30070.03, Lot No. AAM211101, HyClone, Logan, UT, USA), L-glutamine (Cat No. 078-00525, Lot No. CTK0297, FUJIFILM Wako Pure Chemical Corporation, Osaka, Japan), 0.06% sodium bicarbonate (Cat No. 191-01305, Lot No. LDG3012, FUJIFILM Wako Pure Chemical Corporation, Osaka, Japan), and penicillin–streptomycin mixed solution (100 U/mL penicillin and 100 µg/mL streptomycin, Cat No. 09367-34, Lot No. L7N3011, Nacalai Tesque, Inc., Kyoto, Japan) under an atmosphere of 5% CO_2_/95% humidified air at 37 °C. Culture of myocytes and glucose uptake assay and biochemical analyses were conducted essentially as described previously [10,21] with slight modifications [22,23]. L6 myoblasts (5 × 10^4^ cells/well) were subcultured into Multicell Plate for Cell/Tissue Culture 24F with a lid (Cat No. MS-80240, Lot No. 704P8061, Sumitomo Bakelite Co. Ltd., Tokyo, Japan) and grown to 90% confluency for three days in 0.4 mL of 10% FBS/DMEM. Next, the cells were cultured and differentiated to myotubes in 2% FBS/DMEM for one week. The fresh medium was replaced every two days. Differentiated L6 myotubes were finally incubated in filter-sterilized Krebs–Henseleit–HEPES buffer (KHH buffer) (1.2 mM MgSO_4_∙7H_2_O, 7.3 mM NaH_2_PO_4_∙2H_2_O, 47.6 mM KCl, 118 mM NaCl, 3.3 mM CaCl_2_∙2H_2_O, 25 mM NaHCO_3_, pH 7.4) containing 0.1% bovine serum albumin (BSA, fatty acid free), 10 mM HEPES, and 2 mM sodium pyruvate) for 2 h. TXF for treatment was dissolved in dimethyl sulfoxide (DMSO, Cat No. 046-21981, Lot No. TLL1855, FUJIFILM Wako Pure Chemical Corporation, Osaka, Japan) and diluted in KHH buffer containing 11 mM (198 mg/dL) glucose (final concentration of DMSO: 0.1%). Similarly, KHH buffer containing 11 mM glucose for the control (0 µM TXF) was prepared with 0.1% DMSO alone. The myotubes were then incubated for 4 h in KHH buffer containing glucose without or with 50–200 µM taxifolin (TXF) (Cat No. A10893, Lot No. 480-18-2, Adooq Bioscience, Irvine, CA, USA) in the dose-response study. Likewise, the myotubes were incubated for 4 h without or with 25 µM LY294002 (Cat No. 125-04863, Lot No. WDR2373, FUJIFILM Wako Pure Chemical Corporation, Osaka, Japan) as an Akt inhibitor or 10 µM Compound C (dorsomorphin) (Cat No. 040-33753, Lot No. CTF2872, FUJIFILM Wako Pure Chemical Corporation, Osaka, Japan) as an AMPK inhibitor, in the absence or presence of 100 µM TXF. An Akt activator, human recombinant insulin (Cat No. 099-06473, Lot No. LKK2017, FUJIFILM Wako Pure Chemical Corporation, Osaka, Japan) and an AMPK activator, AICAR (5-aminoimidazole-4-carboxamide 1-β-D-ribofuranoside, Cat No. 015-22531, Lot No. LKF0057, FUJIFILM Wako Pure Chemical Corporation, Osaka, Japan) were treated to the cells as the positive control. The differences in the glucose concentrations in the KHH buffer before and after culture were determined with a Glucose CⅡ-Test Kit (Cat No. 439-90901, Lot No. TM341, FUJIFILM Wako Pure Chemical Corporation, Osaka, Japan) and a microplate reader SPARK™ 10M (TECAN, Männedorf, Switzerland) at 505 nm. The amount of glucose consumed was calculated. Similar experiments were conducted at least three times in myotubes differentiated from myoblasts with different passage numbers to confirm the reproducibility of results.

### 2.2. Protein Extraction from L6 Myotubes

L6 myoblasts (5 × 10^5^ cells/well) were subcultured into Nunc EasYDishes 60 mm type (Cat No. 150462, Lot No.8224746, Thermo Fisher Scientific, Ltd., Waltham, MA, USA) and grown to 90% confluency for three days in 3 mL of 10% FBS/DMEM. Then, the cells were cultured to form myotubes in 2% FBS/DMEM for one week. The fresh medium was replaced every two days. After L6 myotubes were kept in KHH buffer for 2 h, the myotubes were then incubated in KHH buffer containing 11 mM glucose without or with 100 µM TXF suspended in 0.1% DMSO for 15–60 min. In the same way, KHH buffer containing 11 mM glucose for control (0 µM TXF) was prepared with 0.1% DMSO alone. To extract protein, the cells were collected from the dishes into RIPA buffer with Protease Inhibitor Cocktail (Cat No. 08714-04, Lot No. L8B4717, Nacalai Tesque, Kyoto, Japan) containing 1% (*v/v*) Phosphatase Inhibitor Cocktail (Cat No. 07575-51, Lot No. L7E1314, Nacalai Tesque, Kyoto, Japan) and sonicated for 5 s on ice. The cell lysate was centrifuged at 10,000× *g* for 10 min at 4 °C. The supernatant was collected as a protein extract. The protein extracts for Akt, AMPK, and their phosphorylation detections were mixed at 1:1 with 4% SDS, incubated at 100 °C for 3 min and stored at −80 °C until use.

### 2.3. Protein Extraction of Plasma Membrane from L6 Myotubes 

The plasma membrane and post-plasma membrane fractions were obtained by the methods described by Nishiumi and Ashida [24] with slight modifications. To prepare the plasma membrane and post-plasma membrane fraction, the 100 µM TXF-treated L6 myotubes were harvested with buffer A (50 mM Tris-HCl (pH 8.0), 0.5 mM dithiothreitol (DTT, Cat No. 048-29224, Lot No. LKF6898, FUJIFILM Wako Pure Chemical Corporation, Osaka, Japan), 1% protease inhibitor cocktail (Cat No. 25955-24, Lot No. L7M2862, Nacalai Tesque, Kyoto, Japan), 1% phosphatase inhibitor cocktail (Cat No. 07575-51, Lot No. L7E1314, Nacalai Tesuque, Kyoto, Japan) containing 0.1% (*v/v*) IGEPAL CA-630 (Cat No.198596, Lot No. M8262, MP Biomedicals, Santa Ana, CA, USA), and homogenized with a 27G needle (Cat No. NN-2719S, Lot No. 170510C, Terumo Corporation, Tokyo, Japan). Each homogenate was centrifuged at 1000× *g* for 10 min at 4 °C. The supernatant was placed on ice for 1 h and centrifuged at 16,000× *g* for 20 min at 4 °C. The obtained supernatant was collected as the post-plasma membrane fraction. The precipitate was suspended in IGEPAL CA-630-free buffer A, and centrifuged at 1000× *g* for 10 min at 4 °C. The precipitate obtained was resuspended in buffer A containing 1.0% (*v/v*) IGEPAL CA-630, stood on ice for 1 h after mixing using the needle, and centrifuged at 16,000× *g* for 20 min at 4 °C. The supernatant was collected as the plasma membrane fraction [21]. The protein extracts of post-plasma membrane and plasma membrane were mixed at 1:1 with 4% SDS and incubated overnight at 4 °C to avoid aggregate formation before use.

### 2.4. Western Blotting

The protein concentrations of each supernatant were evaluated using Pierce™ BCA Protein Assay Kit (Cat No. 23225, Lot No. SH251392, Thermo Fisher Scientific Ltd., Waltham, MA, USA) and a microplate reader (SPARK™ 10M, TECAN, Männedorf, Switzerland) at 562 nm. Equal amounts of protein (15 µg/lane) were loaded onto 10% polyacrylamide gels, separated by electrophoresis (100V), and transferred to Immuno-Blot^®^ PVDF membranes for protein blotting (Cat No. 1620177, Lot No. 09979A09, BIO-RAD, CA, USA) (35 V, 5 h). The membranes were incubated in 5% (*w/v*) BSA/Tris buffered saline with Tween 20 (BSA/TBST) at room temperature for 1 h. After the incubation, the membranes were incubated with primary antibodies: p-AMPK (Thr 172) rabbit antibody (Cat No. 2531, Lot No. 0013, Cell Signaling Technology, Beverly, MA, USA), p-Akt (Ser 473) rabbit antibody (Cat No. 9271, Lot No. 0014, Cell Signaling Technology, Beverly, MA, USA), AMPK rabbit antibody (Cat No. 2532, Lot No. 0019, Cell Signaling Technology, Beverly, MA, USA), Akt rabbit antibody (Cat No. 9272, Lot No. 0025, Cell Signaling Technology, Beverly, MA, USA), GLUT4 (1F8) mouse antibody (Cat No. 2213, Lot No. 0006, Cell Signaling Technology, Beverly, MA, USA), and Na^+^/K^+^-ATPase rabbit antibody (Cat No. 3010, Lot No. 0004, Cell Signaling Technology, Beverly, MA, USA) for 16 h at 4 °C. The membranes were then washed three times with TBST and incubated with secondary antibodies: anti-rabbit IgG HRP linked whole antibody from donkey (Cat No. NA934V, Lot No. 13601187, GE Healthcare UK Ltd., Buckinghamshire, UK) or anti-mouse IgG HRP linked whole antibody from sheep (Cat No. NA931V, Lot No. 16805696, GE Healthcare UK Ltd., Buckinghamshire, UK), for 1 h at room temperature. After washing the membrane three times with TBST, the blots on the membrane were developed by Amersham™ ECL™ Western Blotting Detection Reagent (Cat No. RPN2106, Lot No. 13980045, GE Healthcare UK Ltd., Buckinghamshire, UK) and analyzed with ChemiDoc™ XRS+ with Image Lab™ Software (Cat No. 1708265J1NPC, BIO-RAD, CA, USA). These processes were conducted essentially based on the previous report [21] with proper modifications.

### 2.5. Animal Experiments

KK-A^y^/TaJcl (KK-A^y^/Ta) mice (male, four weeks old, 13 mice, Lot No. A200401-52-52, CLEA Japan, Inc., Tokyo, Japan) and C57BL/6JJcl (C57BL/6J) mice (male, four weeks old, eight mice, Lot No. A160401-53-01, CLEA Japan, Inc., Tokyo, Japan) were purchased from CLEA Japan Inc., Tokyo, Japan. KK-A^y^/Ta mice were adopted as type 2 diabetes (T2D) model animals and C57BL/6J mice as non-diabetic normal ones, since the KK-A^y^/Ta strain is derived from C57BL/6J strain. The mice were housed in an individual cage, maintained in a controlled environmental room (a temperature of 22 °C, a relative humidity of 60% and an 8:00–20:00 light/20:00–8:00 dark cycle). All mice were supplied with a standard mouse chow CRF-1 (Oriental Yeast Co., Tokyo, Japan) and water ad libitum for the entire duration of the study. After acclimation for one week, the KK-A^y^/Ta mice (five weeks old) were divided into two groups: diabetic control group (CNT, seven mice) and TXF orally administered group at 30 mg/kg body weight/day (TXF, six mice), with similar blood glucose levels and body weights (0 week). TXF was suspended in 0.5% (*w/v*) carboxymethyl cellulose sodium salt (CMC; Cat No. 039-01335, Lot No. CTM4818, FUJIFILM Wako Pure Chemical Corporation, Osaka, Japan) solution and orally given by a sonde once a day in the morning for four weeks at a dose of 0.9 mg/0.3 mL/30 g body weight/day = 30 mg/10 mL/kg body weight/day. The non-diabetic normal group (NOR, eight mice) and diabetic control group (CNT) were given 0.5% (*w/v*) CMC solution alone for four weeks. The animal experiment described here was carried out in accordance with the guideline for the Animal Experiments of Utsunomiya University Animal Research Committee and was approved by this committee (Ethic Approval Number: A15-0017).

### 2.6. Time-Dependent Evaluation of Blood Glucose Levels

To evaluate the effect of TXF on blood glucose levels, the blood from the tail vein of mouse was collected once a week for four weeks. The mice were fasted for 90 min from the oral administration at 9:00, but allowed free access to water until blood collection. Blood (5 µL) was collected from tail vein, bursted in 20 µL distilled water, then 20% (*w/v*) trichloroacetic acid (TCA) (Cat No. T9159, Lot No. 040M1683V, Sigma-Aldrich Co. LLC, St. Louis, MO, USA) aqueous solution (25 µL) was added and kept on ice. The mixture was centrifuged at 12,000× *g* for 5 min at 4 °C. The supernatant was collected for determination of the blood glucose levels with a Glucose CⅡ-Test Kit (Cat No. 439-90901, Lot No. TM341, FUJIFILM Wako Pure Chemical Corporation, Osaka, Japan) and a microplate reader SPARK™ 10M (TECAN, Männedorf, Switzerland) at 505 nm, in every week for four weeks. 

### 2.7. Dissection

At 270 min (total 360 min) fasting after the final blood collection from the tail vein at the fourth week, whole blood was drawn from the inferior vena cava of the mice (nine weeks old) anesthetized with isoflurane (Lot No. 158AMA, Pfizer Japan Inc., Tokyo, Japan) and then placed into a heparin-coated tube on ice. The whole blood was centrifuged at 1200× *g* for 20 min at 4 °C to isolate plasma. Levels of glucose, insulin, triglyceride, total cholesterol, uric acid, and adiponectin in the plasma were quantified with the Glucose CⅡ-Test Kit (Cat No. 439-90901, Lot No. TM341, FUJIFILM Wako Pure Chemical Corporation, Osaka, Japan), Morinaga Ultra Sensitive Mouse Insulin ELISA Kit (Cat No. M1104, Lot No. 17SEUMI450, Morinaga Institute of Biological Science, Inc., Kanagawa, Japan), Triglyceride E-Test Kit (Cat No. 432-40201, Lot No. TL635, FUJIFILM Wako Pure Chemical Corporation, Osaka, Japan), Cholesterol E-Test Kit (Cat No. 439-17501, Lot No. TL630, FUJIFILM Wako Pure Chemical Corporation, Osaka, Japan), Uric Acid C-Test Kit (Cat No. 437-17301, Lot No. TG917, FUJIFILM Wako Pure Chemical Corporation, Osaka, Japan), and Mouse Adiponectin/Acrp30 Quantikine^®^ ELISA Kit (Cat No. MRP300, Lot No. P110979, R&D systems, Inc., Minneapolis, MN, USA), respectively. These absorbances were read at 450 nm (insulin and adiponectin), 505 nm (glucose), 555 nm (uric acid), and 600 nm (triglyceride and total cholesterol) using a microplate reader SPARK™ 10M (TECAN, Männedorf, Switzerland). HOMA-IR, an index of insulin resistance, was calculated from plasma glucose and insulin concentrations, as described previously [25,26]. 

### 2.8. Statistical Analysis

All data are represented as the mean values ± SEM. Data on the glucose uptake and the protein expression in L6 myotubes were analyzed by one-way ANOVA with post-hoc Tukey–Kramer’s multiple comparisons test. Data on levels of blood glucose and plasma components were analyzed by one-way ANOVA with post-hoc Dunnett’s multiple comparisons test. Statistical significance was defined as * *p* < 0.05 and ** *p* < 0.01. All statistical analyses were interpreted using the Prism 6 program for Mac OS X (GraphPad Software, San Diego, CA, USA).

## 3. Results

### 3.1. Effect of Taxifolin in Cultured Myocytes

#### 3.1.1. Glucose Uptake in L6 Myotubes and Effect of Taxifolin

**Figure 1:**
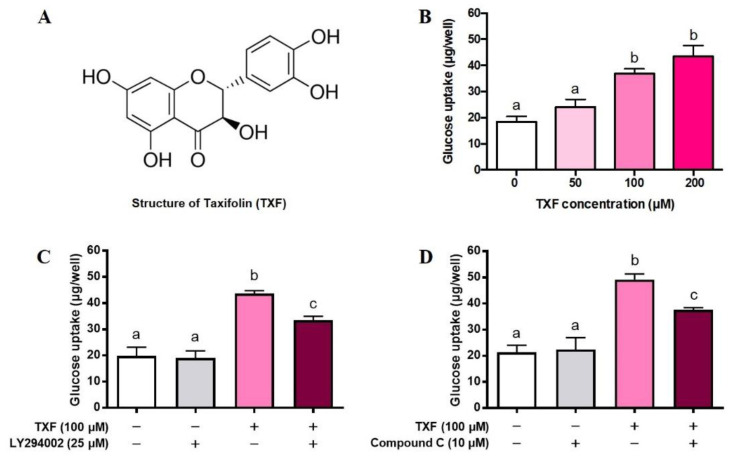
Structure of taxifolin (**A**), dose-dependent effect of taxifolin (**B**), effects of LY294002 (**C**), and Compound C (**D**) on glucose uptake in cultured L6 myotubes for 4 h. Each value represents the mean ± SEM for 6 wells of a 24-multiwell plate. Values not sharing a common letter are significantly different at *p* < 0.05 by Tukey–Kramer multiple comparisons test.

First, we investigated the effect of TXF (Figure 1A) on GU for 4 h in cultured L6 myotubes under the condition of insulin absence. TXF dose-dependently increased GU up to 200 µM, and its increasing effect was significant at 100 and 200 µM (Figure 1B). Selecting the concentration of 100 µM of TXF, we next examined whether or not inhibitors of PI3K (LY294002) and AMPK (Compound C) could cancel the stimulatory action of TXF on GU in L6 myotubes. LY294002 was found to partially but significantly suppress the TXF-induced increase in GU at 25 µM where LY294002 itself exerted no influence on the basal GU in L6 myotubes (Figure 1C), suggesting an involvement of PI3K and hence Akt in the stimulatory action of TXF on GU. Likewise, Compound C partially but significantly suppressed the TXF-induced increase in GU at 10 µM where Compound C itself exerted no influence on the basal GU in L6 myotubes (Figure 1D). These results suggest that TXF upregulates simultaneously and independently both PI3K/Akt and AMPK signaling pathways in GU by L6 myotubes in the absence of insulin. However, either LY294002 alone (Figure 1C) or Compound C alone (Figure 1D) failed to completely suppress the TXF-induced increase in GU to the basal levels at no TXF and no inhibitor treatments; GU was still significantly higher in the concurrent treatments of TXF and each inhibitor, suggesting a possible involvement of other signaling pathway(s) in TXF action on GU in L6 myotubes.

#### 3.1.2. Phosphorylation and Activation of Akt and AMPK by Taxifolin in L6 Myotubes

**Figure 2:**
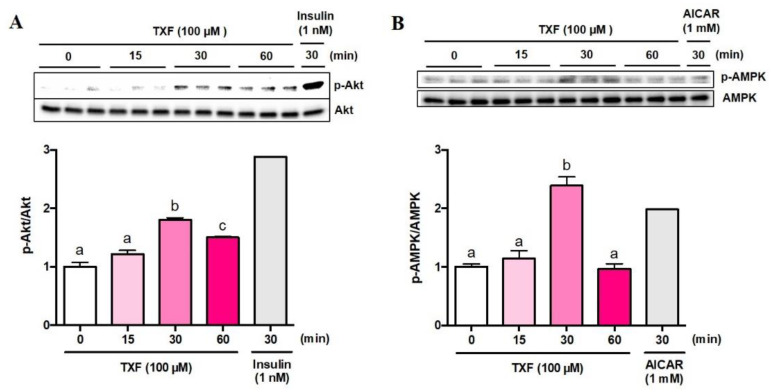
Time-dependent changes in phosphorylation of Akt (**A**) and AMPK (**B**) by TXF (100 µM) in L6 myotubes. Each value represents the mean ± SEM for 3 dishes (60 mm in diameter). Insulin (*n* = 1) and AICAR (*n* = 1) were used as positive controls for Akt and AMPK pathways, respectively, and excluded from statistical analyses. Values not sharing a common letter were significantly different at *p* < 0.05 by the Tukey–Kramer multiple comparison test.

Next, we tried to confirm phosphorylation (=activation) of Akt (p-Akt) and AMPK (p-AMPK) in L6 myotubes by western blot analyses. The ratios of p-Akt to total Akt (p-Akt/Akt) (Figure 2A) and p-AMPK to total AMPK (p-AMPK/AMPK) (Figure 2B) significantly peaked at 30 min after treatment with TXF, although the p-Akt/Akt ratio was still significantly higher than that of the basal level at 60 min after TXF treatment (Figure 2A).

#### 3.1.3. Translocation of GLUT4 to Plasma Membrane by Taxifolin in L6 Myotubes

**Figure 3:**
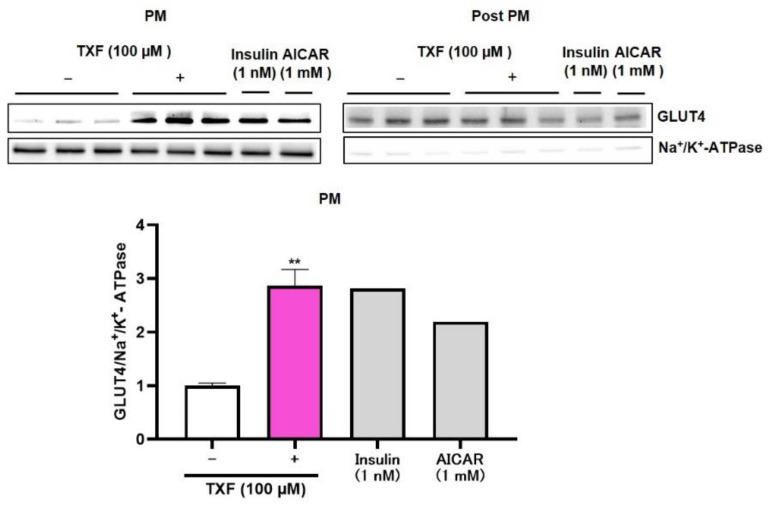
Effect of taxifolin on translocation of GLUT4 to plasma membrane after 30 min exposure of TXF (0 and 100 µM) to L6 myotubes. Each value represents the mean ± SEM for 3 dishes (60 mm in diameter). Insulin (*n* = 1) and AICAR (*n* = 1) were used as positive controls and excluded from statistical analyses. ** Statistically significant from no TXF treatment at *p* < 0.01 by the two-tailed Student’s *t*-test.

As TXF was found to induce phosphorylation (=activation) of both Akt and AMPK in L6 myotubes, we finally confirmed the translocation of GLUT4 to plasma membrane from cytosol of L6 myotubes by western blot analysis after 30 min exposure of TXF to L6 myotubes (Figure 3). Na^+^/K^+^-ATPase was adopted as muscle plasma membrane (PM) marker. Insulin and AICAR were used as positive controls for the Akt and AMPK pathways, respectively. As shown in Figure 3, PM fraction was almost completely isolated from Post PM fraction, since the clear existence of Na^+^/K^+^-ATPase was recognized in the PM fraction, while that in the Post PM fraction was extremely faint. In the PM fraction, TXF treatment significantly raised GLUT4/Na^+^/K^+^-ATPase ratio three times as high as that of no TXF treatment, and this increase in GLUT4 translocation to the plasma membrane was considered to promote GU in L6 myotubes.

### 3.2. Effect of Taxifolin in T2D Model KK-A^y^/Ta Mice

#### 3.2.1. In Vivo Effect of Taxifolin on Food Intake and Body Weight Gain in T2D Model Mice

From the in vitro results obtained from cultured L6 myotubes, TXF was suggested to be capable of promoting glucose transfer from the blood stream into muscle tissues via GLUT4, leading to the hypoglycemic effect in vivo. Therefore, we decided to examine whether or not TXF has a hypoglycemic effect in T2D model KK-A^y^/Ta mice. As shown in Table 1, despite the fact that all the mice were the same age, initial and final body weights, body weight gain, and food intake for four weeks were significantly higher in the diabetic control (CNT) mice than in the normal (NOR) ones. TXF administration exerted no significant influences on these values compared with CNT mice, indicating that hypoglycemic effect of TXF, if any, might not be based on reduced food intake but on its pharmacological action.

#### 3.2.2. In Vivo Effect of Taxifolin on Plasma Glucose, Insulin, and HOMA-IR in T2D Model Mice

**Figure 4:**
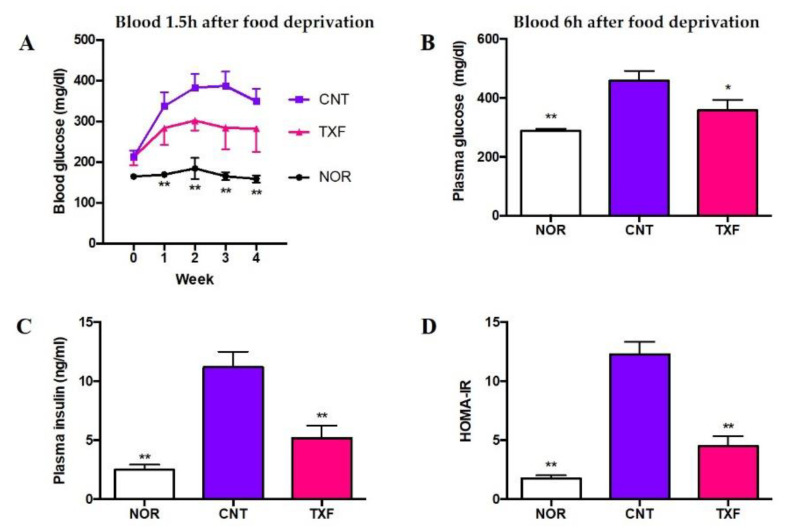
Effect of taxifolin on time-dependent changes in blood glucose levels (**A**), and on fasting plasma glucose (**B**), insulin (**C**), and HOMA-IR (**D**) levels in T2D model KK-A^y^/Ta mice. Changes in blood glucose levels after 1.5 h fasting were measured every week for 4 weeks (**A**). At the end of the experimental period of four weeks, mice were fasted for 6 h, whole blood was collected from each mouse, and plasma sample was prepared. Then, plasma glucose (**B**) and insulin (**C**) levels were determined, and finally HOMA-IR (**D**) was calculated. Each value represents the mean ± SEM for eight (Nondiabetic Normal; NOR), seven (Diabetic Control; CNT), or six (Diabetic Taxifolin-treated; TXF) mice. *^,^ ** Significantly different from the CNT group at * *p* < 0.05 and ** *p* < 0.01, respectively, by Dunnett multiple comparisons test.

Figure 4 shows the effects of taxifolin on time-dependent changes in blood glucose levels for four weeks (A) and fasting plasma levels of glucose (B), insulin (C), and HOMA-IR (D) at the end of the experimental period. To monitor changes in blood glucose levels, blood samples (5 µL) were obtained from the tail vein after mild fasting for 1.5 hours (1.5 h) every week and hemolyzed in water. Glucose in the supernatant after protein precipitation was determined with a commercial kit. As shown in Figure 4A, blood glucose levels in diabetic control mice (CNT) significantly increased up to fourth week compared with those in normal mice (NOR). TXF appeared to suppress the increase, but the differences were not significant from CNT due to the dispersion of data, probably due to the mild fasting period. Thus, we decided to collect whole blood after fasting for six hours (6 h) and obtained plasma at the fourth week of feeding. Plasma levels of glucose (B), insulin (C) and HOMA-IR (D) increased significantly in diabetic control mice (CNT) as compared with normal ones (NOR), and all these increases were significantly suppressed by TXF administration in T2D model KK-A^y^/Ta mice (TXF).

#### 3.2.3. In Vivo Effect of Taxifolin on Plasma Lipids, Uric Acid, and Adiponectin in T2D Model Mice

**Figure 5:**
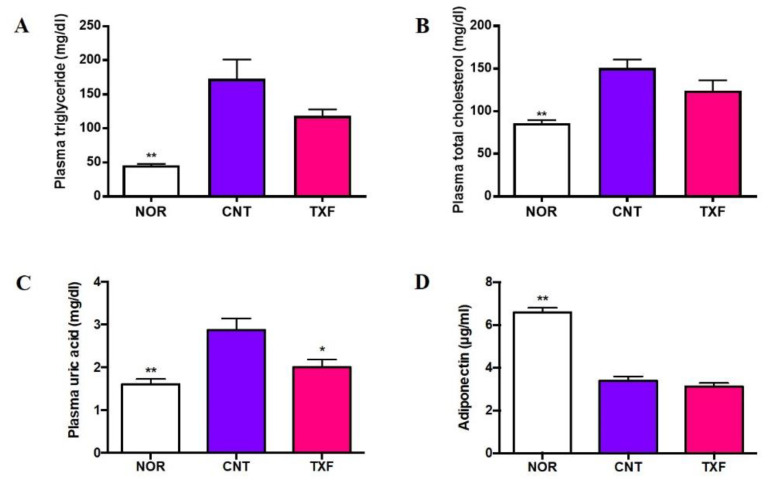
Effect of taxifolin on plasma levels of triglyceride (**A**), total cholesterol (**B**), uric acid (**C**) and adiponectin (**D**) in T2D model KK-A^y^/Ta mice. At the end of experimental period of four weeks, mice were fasted for 6 h, whole blood was collected from each mouse and plasma sample was prepared. Each value represents the mean ± SEM for eight (Nondiabetic Normal; NOR), seven (Diabetic Control; CNT) or 6 (Diabetic Taxifolin-treated; TXF) mice. *, ** Significantly different from the CNT group at * *p* < 0.05 and ** *p* < 0.01, respectively, by Dunnett multiple comparisons test.

Figure 5 shows the effect of taxifolin on the plasma levels of triglyceride (A), total cholesterol (B), uric acid (C), and adiponectin (D) 6 h after fasting at the end of the experimental period of four weeks. Plasma triglyceride and total cholesterol levels were significantly elevated in the diabetic state (CNT) compared with those in the normal state (NOR), but these elevations were not significantly suppressed (*p* < 0.1) by oral administration of taxifolin (TXF) (Figure 5A,B). The plasma uric acid level was significantly higher in the diabetic state (CNT) than in the normal state (NOR), confirming the occurrence of hyperuricemia in T2D model KK-A^y^/Ta mice as previously reported [19], and TXF was found to significantly suppress this hyperuricemia (Figure 5C) as well as hyperglycemia (Figure 4B). In contrast, adiponectin, which is known to activate muscle AMPK [27], significantly decreased in T2D model KK-A^y^/Ta mice (CNT) in comparison with normal ones (NOR), and TXF administration exerted no influence on this reduction (TXF) (Figure 5D).

## 4. Discussion

**Figure 6:**
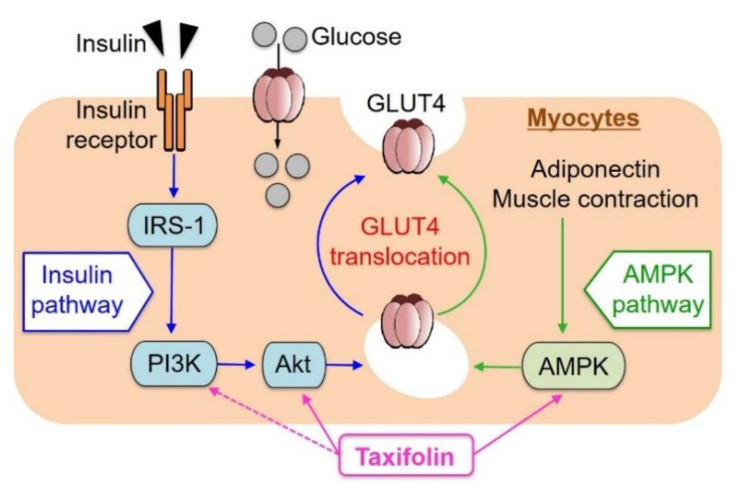
Schematic representation for possible factors involved in glucose uptake by myocytes from extracellular glucose pools.

In the present studies, TXF was demonstrated to dose-dependently increase GU in cultured L6 myotubes via both PI3K/Akt and AMPK signaling pathways such as resveratrol [21]. The activation of each pathway independently promoted GLUT4 translocation to the plasma membrane (Figure 6). In contrast, aspalathin [11] and enterolactone [28] were found to promote GU in L6 myotubes via the AMPK signaling pathway without involvement of the PI3K/Akt pathway, suggesting that the effects of phytochemicals on GU in myocytes might depend on differences in their chemical structures. Piceatannol, a natural analogue and metabolite of resveratrol, has been demonstrated to promote GU via GLUT4 translocation to the plasma membrane by immunocytochemistry in L6 myoblasts transfected with a glut4 cDNA-coding vector as well as western blot analysis in L6 myotubes [29]. Thus, such an immunocytochemical study in L6 myotubes treated with TXF is interesting and desirable aside from western blot analysis, if possible.

Since GLUT4 translocation to the plasma membrane is essential to incorporate glucose into the muscle cells and tissues from outside pools such as medium or buffer in vitro and blood stream in vivo, TXF was considered to be capable of alleviating insulin resistance. In fact, TXF could suppress hyperglycemia in KK-A^y^/Ta mice (Figure 4B) accompanying insulin resistance (Figure 4D). Thus, further studies are required to clarify whether or not TXF promotes AMPK phosphorylation in muscle tissues of KK-A^y^/Ta mice like that reported in the gastrocnemius muscle of T2D model db/db mice that were administered with daidzein [30] because the increase in the AMPK phosphorylation-GLUT4 translocation axis may work even under the state of insulin resistance, thus leading to an antihyperglycemic effect of TXF.

So far, we have not conducted in vitro experiments with concurrent additions of insulin and TXF in L6 myotubes. Ideally, we need to make insulin-resistant L6 myotubes, and then we can conduct in vitro experiments with concurrent additions of insulin and TXF. In our separate in vivo experiment, however, oral administration of daidzein, an isoflavone, to T2D model db/db mice with high serum insulin levels and strong insulin resistance was found to increase the activated (=phosphorylated) AMPK (p-AMPK) in gastrocnemius muscle tissues, and to suppress the increase in blood glucose levels in vivo [30]. In the present in vivo experiment, we did not measure p-AMPK and p-Akt in the skeletal muscle tissues because the timing of skeletal muscle dissection is difficult in in vivo experiments. This kind of experiment is considered to be conducted separately by adopting small numbers of animals. Nonetheless, we believe that even under a high insulin state in the blood (plasma/serum), TXF probably can activate at least AMPK and hence contribute to the reduction in blood glucose levels in vivo such as daidzein [30].

In the present study, the plasma adiponectin level significantly reduced in diabetic control KK-A^y^/Ta mice compared with normal ones, and TXF exerted no influence on the reduction (Figure 5D). TXF might activate AMPK instead of adiponectin (Figure 6). In other T2D model ob/ob mice, the serum adiponectin level also significantly reduced in comparison with the normal ones. In contrast, aspalathin that activated AMPK without affecting Akt in L6 myotubes recovered this reduction in serum adiponectin to the normal level [11]. TXF activated both Akt and AMPK in L6 myotubes, while aspalathin activated only AMPK in L6 myotubes. The reasons for these differences between TXF and aspalathin are unclear at present, and should be clarified in future studies including differences in mice strains and effects on AMPK and/or Akt.

Recently, TXF has been reported to prevent postprandial hyperglycemia by inhibiting the activities of carbohydrate hydrolyzing enzymes such as α-amylase in alloxan-induced diabetic rats [31] and sucrase in sucrose-loaded rats [32]. Thus, TXF might partially play a role as a potent inhibitor of α-amylase and/or sucrase, reduce dietary carbohydrate absorption from the intestinal tract, and show its hypoglycemic effect in T2D model KK-A^y^/Ta mice, although this possibility needs to be clarified experimentally.

TXF has also been reported to improve disorders of glucose metabolism and water-salt metabolism in the kidney through the PI3K/Akt signaling pathway in fructose-loaded, spontaneously hypertensive rats (SHR) [33]. Since KK-A^y^/Ta mice are known as a spontaneous animal model for human T2D nephropathy [34,35], it seems to be of particular interest to study whether or not TXF improve kidney dysfunction as well as hyperglycemia through the PI3K/Akt signaling pathway in KK-A^y^/Ta mice in future studies.

Another important finding in the present study is the anti-hyperuricemic effect of TXF in T2D model KK-A^y^/Ta mice. High uric acid (UA) is demonstrated to directly inhibit insulin signaling by insulin receptor modification and induce insulin resistance [18]. As already reported [19], we could confirm the occurrence of both insulin resistance (Figure 4D) and hyperuricemia (Figure 5C) in KK-A^y^/Ta mice, and TXF was found to alleviate these symptoms. TXF was reported to suppress purine body-induced hyperuricemia by inhibiting hepatic xanthine oxidase, a key enzyme for UA production [20]. Studies on detailed mechanisms for the induction of hyperuricemia and its inhibition by TXF are required in KK-A^y^/Ta mice from the aspects of production and excretion of UA [36]. 

In the present study, TXF was suggested to upregulate both PI3K/Akt and AMPK signaling pathways in L6 myotubes. However, it is necessary and interesting to perform combined treatments of LY294002 and Compound C to confirm that their effect is additive and that the pathways work independently of each other. Since a crosstalk between the Akt and AMPK pathways cannot be excluded [37,38], it would also be worth investigating Akt phosphorylation after Compound C treatment and perform a p-AMPK western blot after blocking Akt by LY294002.

Luo et al. indicated that the development of atherosclerosis and inflammation is promoted by uric acid in vivo. Moreover, the lowering of uric acid levels attenuated inflammation via the activation of the AMPK pathway [39]. Kimura et al. have recently reported that the development of atherosclerosis and inflammation are promoted by uric acid in vivo, where the lowering of uric acid levels attenuated inflammation via the activation of the AMPK pathway and provided mechanistic evidence of uric acid lowering therapies for atherosclerosis [40]. As already mentioned, TXF has been found to activate AMPK and alleviate hyperuricemia induced by purine-bodies in mice by inhibiting xanthin oxidase [20]. TXF was also demonstrated to suppress hyperuricemia in T2D model KK-A^y^/Ta mice in the present study (Figure 5C). These findings provide mechanistic evidence of uric acid lowering therapies of TXF for inflammation and atherosclerosis as well as T2D.

As genotoxicity [41] and mutagenicity [42] of TXF have been demonstrated to be very low, this phytochemical is considered to be a highly safe hypoglycemic and hypouricemic biofactor.

## Figures and Tables

**Table 1:** Initial and final body weights, body weight gain, and food intake for four weeks in T2D model KK-A^y^/Ta mice.

Measurement	NOR	CNT	TXF
Initial body weight (g)	19.4 ± 0.2 **	28.4 ± 0.5	28.7 ± 0.8
Final body weight (g)	23.1 ± 0.3 **	37.6 ± 0.9	36.1 ± 1.3
Weight gain (g/4 weeks)	3.7 ± 0.2 **	9.2 ± 0.6	7.4 ± 1.0
Food intake (g/4 weeks)	89.1 ± 1.3 **	162.9 ± 6.0	154.9 ± 9.4

Nondiabetic normal mice (C57BL/6J) and diabetic control (KK-A^y^/Ta) mice received 0.5% sodium carboxymethyl cellulose (CMC) solution alone, and taxifolin (TXF) suspended in CMC was orally given to KK-A^y^/Ta mice once a day for four weeks at a dose of 30 mg/kg body weight/day. C57BL/6J mice were adopted as nondiabetic normal ones, since the KK-A^y^/Ta strain was derived from this strain. Each value represents the mean ± SEM for eight (Nondiabetic Normal; NOR), seven (Diabetic Control; CNT), or six (Diabetic Taxifolin-treated; TXF) mice. ** Significantly different from the CNT group at *p* < 0.01 by Dunnett’s multiple comparison tests.

## Data Availability

Not applicable.

## Abbreviations

AICAR	5-aminoimidazole-4-carboxamide 1-β-D-ribofuranoside
Akt	protein kinase B
AMPK	5’-adenosine monophosphate-activated protein kinase
BSA	bovine serum albumin
DTT	dithiothreitol
DMEM	Dulbecco’s modified Eagle medium
FBS	fetal bovine serum
GLUT4	glucose transporter 4
GU	glucose uptake
HOMA-IR	homeostasis model assessment of insulin resistance
KHH buffer	Krebs–Henseleit–HEPES buffer
PI3K	phosphatidylinositol 3-kinase
TBST	Tris buffered saline with Tween 20
T2D	type 2 diabetes
TXF	taxifolin
UA	uric acid
